# Floral signals evolve in a predictable way under artificial and pollinator selection in *Brassica rapa*

**DOI:** 10.1186/s12862-020-01692-7

**Published:** 2020-09-24

**Authors:** Pengjuan Zu, Florian P. Schiestl, Daniel Gervasi, Xin Li, Daniel Runcie, Frédéric Guillaume

**Affiliations:** 1 Department of Systematic and Evolutionary Botany, University of Zürich, Zollikerstrasse 107, CH-8008 Zürich, Switzerland; 2 Department of Civil and Environmental Engineering, Massachusetts Institute of Technology, 77 Massachusetts Avenue, Cambridge, MA 02139 USA; 3 Department of Plant Sciences, University of California Davis, One Shields Avenue, Davis, CA 95616 USA; 4 Department of Evolutionary Biology and Environmental Studies, University of Zürich, Winterthurerstrasse 190, CH-8057 Zürich, Switzerland

**Keywords:** Adaptive evolution, Artificial selection, *Brassica rapa*, Experimental evolution, Floral scent, G-matrix, Multivariate prediction, Pollinator selection

## Abstract

**Background:**

Angiosperms employ an astonishing variety of visual and olfactory floral signals that are generally thought to evolve under natural selection. Those morphological and chemical traits can form highly correlated sets of traits. It is not always clear which of these are used by pollinators as primary targets of selection and which would be indirectly selected by being linked to those primary targets. Quantitative genetics tools for predicting multiple traits response to selection have been developed since long and have advanced our understanding of evolution of genetically correlated traits in various biological systems. We use these tools to predict the evolutionary trajectories of floral traits and understand the selection pressures acting on them.

**Results:**

We used data from an artificial selection and a pollinator (bumblebee, hoverfly) evolution experiment with fast cycling *Brassica rapa* plants to predict evolutionary changes of 12 floral volatiles and 4 morphological floral traits in response to selection. Using the observed selection gradients and the genetic variance-covariance matrix (G-matrix) of the traits, we showed that the observed responses of most floral traits including volatiles were predicted in the right direction in both artificial- and bumblebee-selection experiment. Genetic covariance had a mix of constraining and facilitating effects on evolutionary responses. We further revealed that G-matrices also evolved in the selection processes.

**Conclusions:**

Overall, our integrative study shows that floral signals, especially volatiles, evolve under selection in a mostly predictable way, at least during short term evolution. Evolutionary constraints stemming from genetic covariance affected traits evolutionary trajectories and thus it is important to include genetic covariance for predicting the evolutionary changes of a comprehensive suite of traits. Other processes such as resource limitation and selfing also need to be considered for a better understanding of floral trait evolution.

## Background

Understanding and predicting the evolutionary responses of phenotypes to selection remains a major challenge in evolutionary biology. This undertaking is not trivial because phenotypes are often complex traits co-evolving with each other underlain by complex genetic architectures. Yet, understanding how such co-evolutionary units evolve under natural selection is important to understand how species may respond to changes in their environment. Flowers are complex organs with enormous diversity in morphology, color and scent, and thus comprise a complex set of interrelated traits. These visual and olfactory components, which characterize the radiation of angiosperms, are recognized to evolve as a means of interaction with their biotic environment [1, 2]. One key driver, the pollinators, has been emphasized to be important for floral trait evolution since long [3, 4]. Yet, only a handful of studies have attempted to predict evolutionary responses of floral traits to pollinator selection [5–9] . Moreover, these studies only examined one or a few morphological traits at a time, whereas interactions of flowers with other organisms are typically mediated by a combination of traits of morphological and/or olfactory nature [10, 11]. A multivariate approach can, therefore, help to unravel the genetic architecture of floral traits and predict their joint evolution.

A great number of empirical studies have documented significant heritability and genetic (co)variance of diverse floral traits [12–15], as well as phenotypic selection acting on them [16–27]. Among those traits, floral scents have started drawing more and more attention. Floral scents are usually highly variable and diverse on all taxonomic levels [28], and many studies have documented natural selection on scent [22–25, 29, 30]. Earlier studies have shown that scent phenotypic variation has a significant heritable genetic component in fast-cycling *Brassica rapa* (20–45%, [15]). In the same species, Gervasi and Schiestl [25] showed in a greenhouse experiment that bumblebee pollinator selection resulted in taller plants with higher UV-reflecting flowers and increased amounts of several floral scents, presumably used by the bees as visual and olfactory signals. However, the selection intensities acting on each trait alone could not fully explain the realized evolutionary changes [25]. Phenotypic trait responses to selection are known to depend on the pattern of genetic variance-covariance among them [31, 32]. In particular, traits that are genetically correlated because of a shared genetic basis (e.g., pleiotropic genes) will indirectly respond to selection on linked traits, which may mask the effect of direct selection on them. Therefore, the targets of direct selection cannot be well characterized unless the selection responses are decomposed into their direct and indirect components. This is best done using a multivariate quantitative genetics framework [31, 33].

Quantitative genetics theory provides a means to make such evolutionary predictions in the form of the multivariate breeder’s equation (or Lande’s equation), △**z** = **Gβ** [31]. Lande’s equation predicts the per-generation change in a set of quantitative traits in a population (△**z**) as the product of their genetic variance-covariance matrix (G-matrix) with the vector of selection gradients acting on them (**β**). The components of Lande’s equation can be estimated from phenotypic and individual pedigree relationship data in an experiment by using the classical tools of quantitative genetics [32, 34]. More importantly, this multivariate approach can help distinguish between the direct and indirect responses to pollinator selection. The direct component of the response to selection is obtained by multiplying the diagonal elements of the G-matrix (*G*_*ii*_, the additive genetic variance of the traits) with the **β** vector, which holds, for a single trait *i*: △*z*_*i*_^direct^ = *G*_ii_*β_*i*_, while its indirect component is the product of the off-diagonal elements of **G** (genetic covariance: *G*_*ij*_) with **β**, summed over all traits *j* ≠ *i*: △*z*_*i*_^indirect^ = ∑*G*_ij_*β_*j*_. The total response is the sum of these two components. Traditionally, the distinction between direct and indirect selection has been made by comparing the selection differential and the selection gradient acting on each trait separately [32]. A selection differential (*S*) is the phenotypic covariance between relative fitness *w* and the trait *z*: *S* = Cov(*w*, *z*), while *β* is the coefficient of regression of relative fitness on the trait value *z*: *β* = *S/V*_*P*_ (with *V*_*P*_ the phenotypic variance) [32]. *S* includes the direct and indirect effects of selection on the trait but it cannot distinguish between them (unless in artificial selection where the directly imposed selection is known). Under this approach, a trait is said to be under direct selection if its selection gradient estimate *β* is significant. Otherwise, the selection represented by a significant selection differential is interpreted as a mix of direct and indirect selection. However, the relative importance of direct and indirect selection can be established when comparing the direct and indirect components of the predicted selection response obtained from Lande’s equation. In that case, if the total predicted response of a trait is opposed to or smaller than its direct component, then evolution of that trait will be said to be constrained by the genetic correlation among the traits. Furthermore, if the observed response is well predicted by the total response and the direct selection component is in the same direction as the observed response, then the trait can be said to be a target of direct selection. Otherwise, if observed and direct responses are opposed, the trait response is more influenced by indirect selection than direct selection and is thus likely evolutionarily constrained. Because these inferences use the G-matrix, they are less influenced by non-genetic causes of association among traits than inferences based on the selection differentials. Finally, one key evolutionary insight we can get from the multivariate quantitative genetic framework presented here is that traits may deviate from their predicted changes under direct selection (**β**) because of indirect selection pressures caused by selection on the other traits and their genetic correlation with them. In other words, traits may deviate from their expected evolutionary trajectory given by **β**, and thus be constrained by genetic correlations with traits under a different set of selection pressures [35, 36].

In this study, we predict floral traits evolution under artificial and pollinator selection from estimates of the G-matrix and the selection gradients of the traits. We tested how the evolutionary trajectory of a trait is affected by genetic correlations among traits by dissecting the total responses to selection into their direct and indirect components. By comparing the direction of the observed trait responses with the directions of their direct and indirect predicted responses, we tested whether traits were targets of direct selection in the pollinator experiment. We also assessed the evolution of genetic architectures (G-matrices) during the artificial selection process. We used data from two forward-in-time experimental evolution experiments that documented genetic co-variation and evolutionary responses in floral traits of fast cycling *Brassica rapa* plants. The G-matrix of the plant population was estimated from a three-generation bi-directional artificial selection experiment on plant height [14]. In that study, tall- and short-plants were selected artificially for building the two directional lines, plus randomly selected plants for an additional control line. Four morphological floral traits and 12 floral volatiles were measured in each generation. Control lines in this experiment were used to estimate the G-matrix. The selection gradients **β** were estimated in four evolutionary experiments: two for the tall- and short-selection lines in the artificial selection experiment mentioned above [14]; the other two from a 9-generation pollinator selection experiment [25]. The pollinator selection experiment was carried out with bumblebees and hoverflies as the selection agents separately. The same set of floral traits were measured, and the parental plants were from the same seed bank as in the artificial-selection experiment.

## Results

### Predictions in the artificial selection experiment

**Fig. 1:**
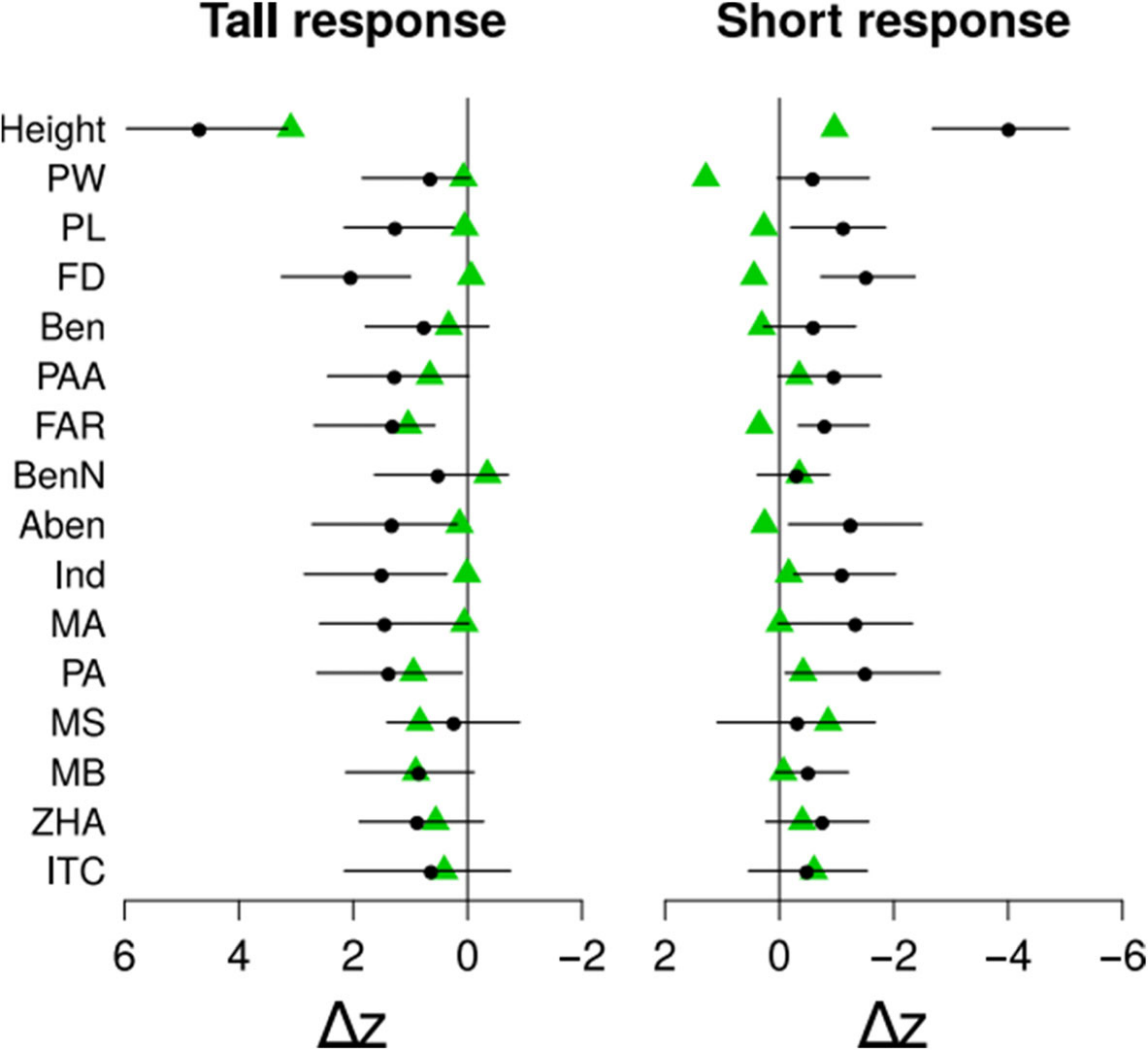
Predicted and observed responses of measured traits to artificial selection. Green triangles are the observed changes. Black dots are the predicted selection responses. The solid horizontal lines indicate the 95% HPD interval of the predictions. Both predicted and observed changes were scaled by the phenotypic standard deviation of the trait. Sample sizes: plant height: 600; flower size traits (PW, PL, FD): 581; volatiles: 579. Trait abbreviations: Plant height (Height), Petal width (PW), Petal length (PL), Flower diameter (FD), Benzaldehyde (Ben), Phenylacetaldehyde (PAA), α-Farnesene (FAR), Benzyl nitrile (BenN), 2-Amino benzaldehyde (Aben), Indole (Ind), Methyl anthranilate (MA), Phenylethyl alcohol (PA), Methyl salicylate (MS), Methyl benzoate (MB), Z-(3)-Hexenyl acetate (ZHA), 1-Butene-4-isothiocyante (ITC)

The direction of the response of plant height, the direct and only target of artificial selection, was correctly predicted in the “tall” and in the “short” treatment (Fig. 1), although the observed responses of plant height were smaller than their predictions in both experiments. Given that all other traits are positively correlated with plant height (see Table S4), their indirect responses are predicted positive for tall lines and negative for short lines. However, observed responses of the flower size traits (petal width, PW; petal length, PL; and flower diameter, FD) were positive in short lines and close to zero in tall lines (Fig. 1). The direction of the correlated responses of floral volatile organic compounds (VOCs) were predicted well in most of the traits in both treatments (Fig. 1), although the predicted responses were not significantly different from zero in about half of the traits because their highest probability density (HPD) intervals overlapped with zero (Fig. 1).

### Predictions in the pollinator evolution experiment

**Fig. 2:**
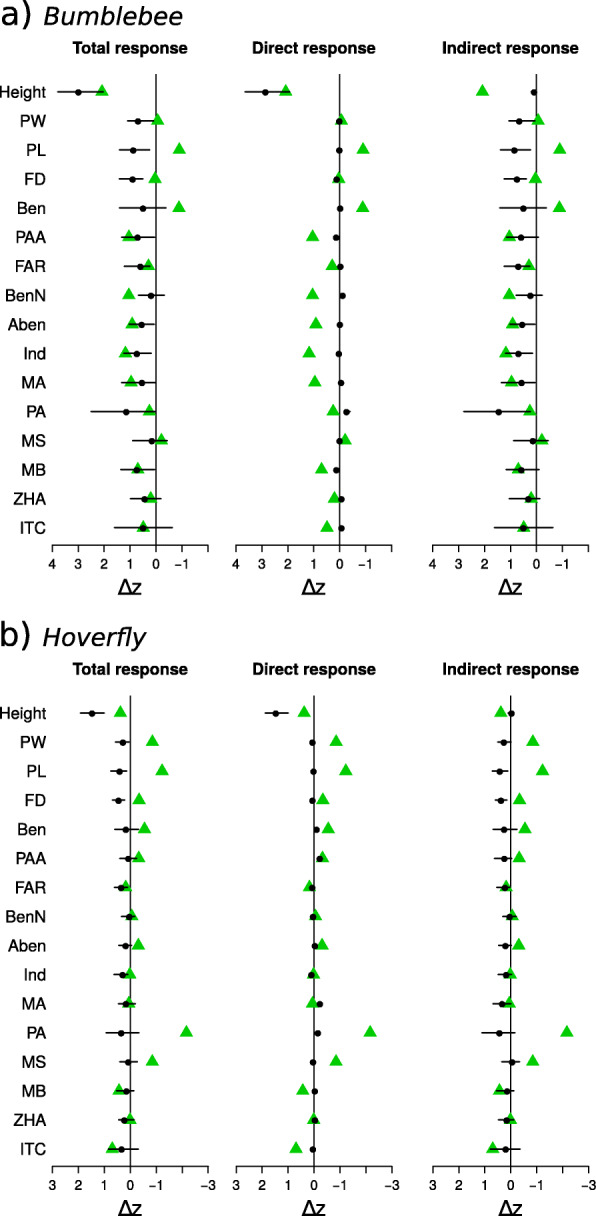
Predicted and observed responses of measured traits to pollinator selection, in the bumblebee (**a**), and hoverfly (**b**) experiments. The total predicted response of each trait is decomposed into its direct and indirect components (see text). Green triangles are the observed changes. Black dots are the predicted selection responses. The solid horizontal lines indicate the 95% HPD interval of the predictions. Both predicted and observed changes were scaled by the phenotypic standard deviation of the trait. Sample sizes pollinator selection: plant height: 524, flower traits: 525, volatiles: 414. Trait abbreviations: Plant height (Height), Petal width (PW), Petal length (PL), Flower diameter (FD), Benzaldehyde (Ben), Phenylacetaldehyde (PAA), α-Farnesene (FAR), Benzyl nitrile (BenN), 2-Amino benzaldehyde (Aben), Indole (Ind), Methyl anthranilate (MA), Phenylethyl alcohol (PA), Methyl salicylate (MS), Methyl benzoate (MB), Z-(3)-Hexenyl acetate (ZHA), 1-Butene-4-isothiocyante (ITC)

In the bumblebee treatment, our predictions overestimated the evolutionary changes of flower morphological traits and correctly estimated the response of plant height and nine of the VOCs. Although the responses of the nine scent compounds were in the same direction and within the HPD intervals of their predictions, only seven of the predicted responses were significantly different from zero (phenylacetaldehyde, PAA; α-farnesene, FAR; 2-amino benzaldehyde, Aben; indole, Ind; methyl anthranilate, MA; phenylethyl alcohol, PA; methyl benzoate, MB; Fig. 2a; Table S6).

The response decomposition analysis showed that most direct responses were much smaller and often in the wrong direction relative to the observed responses. The direct response was correctly predicted as positive for plant height and flow diameter (FD) among morphological traits, and phenylacetaldehyde, 2-amino benzaldehyde, indole, and methyl benzoate among VOCs (Fig. 2a). Selection gradients (direct responses) and observed responses were thus correctly aligned for those traits, making them good candidates for targets of direct selection, although the response of flow diameter was smaller than predicted. In contrast, the indirect components of the predicted responses were all positive and had much larger HPD intervals, often overlapping with zero (although not for morphological traits, α-farnesene, 2-amino benzaldehyde, indole, methyl anthranilate, and phenylethyl alcohol). Therefore, the indirect VOC responses compensated for negative direct selection responses in α-farnesene, methyl anthranilate, and phenylethyl alcohol (for those traits with correct and significant total predicted responses).

In the hoverfly treatment, most VOC trait responses were small compared to the bumblebee treatment (Fig. 2b). Evolutionary predictions were mostly not different from zero except for plant height, flower morphology, α-farnesene and indole, although predicted responses of flower traits were opposed to their observations. Of the 12 VOCs, only α-farnesene’s response was within its prediction’s HPD interval and different from zero (Fig. 2b; Table S6). From the decomposition of trait responses, α-farnesene would be the most likely candidate for a trait under direct hoverfly selection. Other traits also had observed and direct responses aligned but their predictions were not significantly different from zero (benzaldehyde, 2-amino benzaldehyde, phenylacetaldehyde, phenylethyl alcohol, 1-butene-4-isothiocyanate) or did not include the observed change within their HPD (plant height, indole) (Table S6). The VOC indirect response components were all positive and not significantly different from zero (although not for α-farnesene). All morphological traits’ indirect responses were positive and significant (Fig. 2b; Table S6).

### Effects of genetic covariance on predicting evolutionary trajectories

**Fig. 3:**
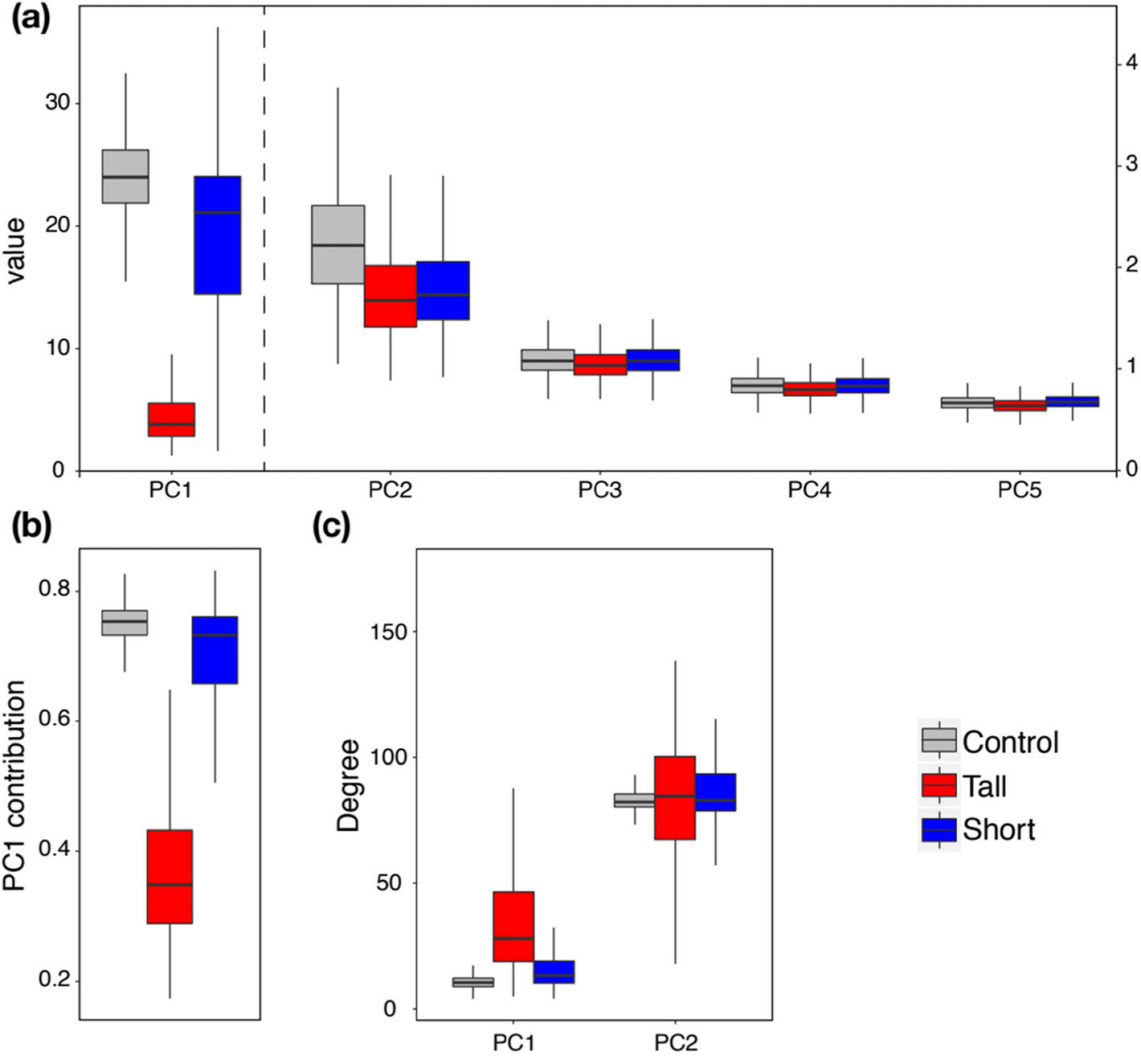
Comparison of the size and orientation of the major and five first eigenvectors (PCs) of the G-matrices in the artificial selection experiment. **a** Distribution of the eigenvalues (size) of each PC of the three G-matrices in the control (grey), tall (red), and short (blue) artificial selection experiments. The scale of the *y*-axis is on the left for PC1 and on the right for PC2–5. **b** Contribution of PC1 to the total variation in the 16 traits, measured as the size of PC1 relative to the sum of all PCs. **c** Angle of the first and second PC with the first trait axis (height) in degree. In all cases, variation of all variables stems from the posterior distribution of each G-matrix estimated with MCMCglmm (see Methods and Supporting information)

We measured the overall constraining effect of genetic co-variation on the response to selection by comparing the angle **θ** between the selection response vector (△**z**) and the first PC of **G** (PC1, or ***g***_max_, see methods) with the angle **γ** between △**z** and the selection gradient (**β**). In the tall and short artificial selection experiments, the trait responses were strongly aligned with ***g***_max_, with **θ** angle of 8.9 degree (95% HPD: 5.5, 13.7) and 20.1 degree (95% HPD: 17.5, 23.5), respectively. Given the close association of ***g***_max_ with the first trait axis (height) (Fig. 3c) and thus with the selection gradients under artificial selection, the angle **γ** between △**z** and **β** is 6.8 and 16.7 degree in tall and short, respectively. In contrast, under pollinator selection, △**z** is more aligned with ***g***_max_ than **β**, with **θ** of 50.6 degree (95% HPD: 44.7, 55.0) and 54.3 degree (95% HPD: 52.0, 57.0), when compared to **γ**, equal to 62.96 and 83.2 degree for bumblebee and hoverfly treatments, respectively.

### Evolution of the G-matrix during artificial selection

By examining the G-matrices of the three lines in the artificial selection experiment (**G**_control_, **G**_tall_, and **G**_short_), we found a drastic decrease of the additive genetic variance of height in the tall line, with an estimate around 2.8 cm^2^, compared to the short line, which remained as high as in the control line around 23 cm^2^. This resulted in a large decrease of the contribution of ***g***_max_ (PC1) of **G**_tall_ to the total variance relative to **G**_control_ and **G**_short_ (see Fig. 3a-b, Table S5). The orientation of ***g***_max_ also changed in **G**_tall_, with reduced alignment with the height axis (Fig. 3c). The other eigenvalues and eigenvectors are, however, more constant across lines (Fig. 3a). For instance, the second eigenvector (PC2) is more consistently orthogonal to the height trait axis in the three **G**-matrices (Fig. 3c).

To further compare G-matrices, we used two different approaches from the toolkit of G-matrix comparisons, the random skewers and CPC approaches (Roff et al. 2012 [37], see Methods). Using the random skewers method, we found strong correlations of the mean selection response among matrices, larger than 70% for all three comparisons, although not significantly so between **G**_tall_ and **G**_short_, and very strong similarity between **G**_control_ and **G**_short_ (Table 1). The three G-matrices thus shared a significant portion of their structure. **G**_control_ would predict selection responses similar to **G**_short_ and to a lesser extent to **G**_tall_. Further analysis of the similarity of the size and orientation of the eigenvectors of the G-matrices in the hierarchical analysis (CPC) confirmed the similarity in shape between **G**_control_ and **G**_short_ and the dissimilarity of **G**_tall_ with **G**_control_, and with **G**_short_ to a smaller degree (see Table 1). The G-matrix in the tall lines thus evolved more than in the short lines mostly because of the change in the genetic variance of plant height. **G**_short_ remained closer to the starting G-matrix (**G**_control_) over the course of the experiment.

**Table 1:** Comparisons of the three G-matrices using random skewers and hierarchical analyses (see also Table S3, S4). The random skewers section reports the mean correlation among response vectors of two G-matrices subject to the same set of 10,000 random selection vectors. The hierarchical analysis reports the *P*-values to reject the hypotheses of equality, proportionality, or common principal components (CPC) in favor of *unrelated* matrices. The *P*-values are obtained by randomization (see Methods)

	Random Skewers		Hierarchical		
Paired G	Mean correlation	***P***-value	Equal	Prop.	CPC
**G**_control_ - **G**_tall_	0.734	< 0.01^a^	< 0.005	< 0.005	0.002
**G**_control_ - **G**_short_	0. 987	< 0.002^b^	0.23	0.23	0.32
**G**_tall_ - **G**_short_	0.722	< 0.21^a^	0.19	0.19	< 0.05

^a^: left tail; ^b^: right tail

### Evaluation of the estimation of the **G**-matrices

Permutation tests of G-matrices were conducted to examine whether our G-matrix estimates captured the meaningful biological structure of the data. The results revealed that the majority of the genetic covariance elements (101 out of 120) and additive genetic variances (14 out of 16) in **G**_control_ were significantly different from zero at the level of FDR <  0.05 with 500 permutations and after correcting for multiple testing (false discovery rate: Benjamini & Hochberg, 1995 [38]). In **G**_tall_, 11 variance and 55 covariance elements were significant, and 15 and 72 elements, respectively, in **G**_short_ (Table S4), at the same FDR level. Furthermore, variance estimates had much narrower 95% HPD intervals than covariance estimates (from their posterior distributions, results not shown), as evident in the size of HPD intervals of the direct and indirect components of the selection responses (see Fig. 2).

## Discussion

Total evolutionary trait responses are made of direct and indirect responses. Evolutionary constraints emerge when the two oppose each other. However, constraints may evolve when selection or other evolutionary forces alter the genetic variance and covariance among traits (i.e., change the underlying genetic pleiotropic effects or linkage disequilibrium). It is thus important to evaluate the structure of the G-matrix and its evolution when trying to understand the effects of selection on multiple phenotypic traits. Moreover, being able to compare predicted and realized trait responses allows for a better understanding of the relationship between selection and genetic constraints. In this study, by combining estimates of the ancestral G-matrix of the traits with estimates of the selection gradients acting on them, we could predict the evolutionary response of floral traits subject to two types of selection pressures, artificial and pollinator selection. Importantly, we found that predictions based only on the direct trait responses to selection failed to predict the observed responses and that the observed responses were biased towards the line of least genetic resistance (***g***_max_) of the G-matrix. The pattern of genetic covariation among traits thus strongly affected the outcome of selection in the artificial and pollinator selection experiments. Although this pattern of trait covariation can change during evolution, we further showed that using an ancestral G-matrix, here estimated in the control lines, can lead to accurate evolutionary predictions over just a few generations. This approach allowed us to better understand how pollinators, the selective agents, interact with the complex set of floral traits composed of floral scent and morphology and may influence their evolution.

Overall, bumblebee selection was in favor of taller plants and increased emission of certain floral volatiles, most notably indole (Ind), phenylacetaldehyde (PAA), 2-amino benzaldehyde (Aben), and methyl benzoate (MB) (see also [25]). Those traits had a significant positive direct selection response in the same direction as their observed response making them candidates for direct targets of bumblebee selection. The indirect components of the responses were also all positive, enhancing the total predicted responses, sometimes leading to overshooting of the observed responses. Because of the largely positive genetic correlation of most floral traits with height, it is not surprising to observe positive total selection responses of most traits. In fact, our analysis of direct and indirect responses indicated that the strong increase in many volatiles observed by Gervasi and Schiestl [25] is to some degree a consequence of indirect selection, whereas the response in height is mostly driven by direct direction. Therefore, bumblebees seemed to primarily select for tall plants, and some volatiles, too, although the evolutionary increase in height carried them along. Our analysis also revealed that some of the positive total responses in volatiles may actually be maladaptive because they were opposed to the selection gradient acting on them (e.g., α-farnesene (FAR), benzyl nitrile (BenN), methyl anthranilate (MA), phenylethyl alcohol (PA), z-(3)-hexenyl acetate (ZHA), and 1-butene-4-isothyocyanate (ITC), see Fig. 2a, Table S6). This suggests that bumblebees tended to dislike flowers with increased concentration of those volatiles, but their evolutionary increase was indirectly caused by selection on height and other positively correlated traits that were under positive selection. These positive, non-adaptive responses thus point to the existence of strong evolutionary constraints stemming from the genetic architecture of the traits. Overall, knowledge of the selection gradient, the G-matrix and responses of the traits showed that they evolved in a direction biased towards ***g***_max_, the “line of least resistance” [35], which constrained the evolutionary response away from the selection gradient, although the selection responses of some traits were enhanced by trait covariation.

In contrast to predictions in the bumblebee-pollinated plants, the ones in hoverfly-pollinated plants were largely not different from zero or incorrect. The observed changes were also not consistently in the same direction. This implies that an evolutionary response along one major axis of overall positive trait co-variation is not likely, at least when estimating the co-linearity of the response vector with ***g***_max_ of **G**_control_, and that selection was rather ineffective. Instead, the observed changes are more consistent with altered patterns of trait covariation and drift. Indeed, in the hoverfly-selection experiment, a separate study found very little adaptive evolution in plant traits with the exception of strongly increased autonomous selfing [25]. Thus, increased selfing and the associated reduction of genetic variation [39], possibly altered the G-matrix, leading to the low accuracy of our predictions and the reduced efficiency of pollinator-induced selection. Previous studies in bottlenecked insect populations have shown that rapid changes in the G-matrix are expected in inbred populations (e.g., [40, 41]).

We observed further discrepancies between our evolutionary predictions and observed responses that need to be examined. In particular, the responses of the morphological traits in the artificial selection didn’t show the expected changes of plant height. Plant height did evolve in the correct direction but with a smaller response than expected from the estimate of the additive genetic variance in **G**_control_ (see Table S4). The discrepancy can be caused by a reduction of the genetic variance during the selection experiment, as seen in the tall lines (Table S4). The prediction didn’t take account of those changes. However, the lack of response in the short line is stronger and not likely caused by a reduction of genetic variance, not seen in **G**_short_ (see Table S4). Instead, this selection experiment may have revealed an underlying resource allocation trade-off masked by the apparent positive genetic covariation between plant height and the size of the reproductive organs. This is reminiscent of classical theory on the effect of variation in resource acquisition and allocation on fitness components [42–44], which states that a positive correlation between fitness components can be observed despite an underlying trade-off when individuals vary more in the acquisition than in the allocation of their resources. Variation in resource acquisition among the genotypes may have been pre-existing in the base population of *B. rapa*, and lead to the observed positive correlation between traits pertaining to two fitness components, plant reproduction for flower size traits, and plant somatic growth for plant height. Nevertheless, a resource allocation trade-off may have constrained evolutionary changes of plant height and flower size traits in both the tall and short lines as evidenced by smaller than expected and even opposed changes in flower size traits relative to plant height, and a lower response of plant height.

### The role of genetic covariance in adaptive evolution

Our results are in line with the established expectation that genetic covariance can influence traits’ evolutionary responses by constraining or augmenting their response to selection depending on the relative signs of genetic covariances and selection gradients [31, 33, 45]. This expectation has been rarely directly tested with experimental evolution as we did here (see also [46]). More commonly, empirical studies use estimates of contemporary selection gradients and G-matrices to evaluate the potential for evolutionary constraints, which are present in some cases (e.g., [47–50]) but not in others (e.g., [36, 51, 52]).

The relevance of predictions of evolutionary constraints depends on the constancy of patterns of genetic variance-covariance over time. Our study shows that constancy cannot be assured when selection strongly reduces the genetic variance of a trait, as during artificial selection for taller plants (see also [46, 53, 54]). Yet, using **G**_control_ as an estimate of the ancestral G-matrix allowed us to make correct evolutionary predictions of the direction of selection responses in most cases. Had we used **G**_tall_ in the tall selection experiment, we would have badly underestimated the selection response of plant height and floral scents (results not shown). This illustrates two important points concerning the evolutionary significance of the structure of the G-matrix. First, changes in **G** can happen quickly, over just a few generations, and we have illustrated a rapid change in trait variance caused by selection. Second, despite those changes, estimation of **G** is still useful to make predictions of future trait changes over few generations. This can be useful to predict evolution and adaptation under rapid environmental changes, for instance, because the state of the G-matrix before a change in selection pressures will strongly influence the resulting evolutionary trajectory of a population, as we have shown here.

The evolutionary significance of the structure of the G-matrix is still debated, especially regarding the interpretation of the constraining effects of the main eigenvectors of **G** (especially ***g***_max_). The debate, however, mostly crystallized on inferences of past evolutionary constraints from contemporary estimates of trait variance-covariance patterns. The retrospective use of **G** is questionable knowing how evolutionarily labile are patterns of variance-covariance, an important caveat already emphasized by Turelli [55]. Indeed, many processes may affect the evolution of trait variance and covariances because they depend on variation in allele frequencies in a population. As such, genetic drift [56] and fluctuating selection [57], have been shown to reduce the stability of the G-matrix, while migration [58], correlational selection [56], and mutation [56, 59] can improve its stability (reviewed in [60]). Those changes thus make retrospective use of **G** at the least dangerous, unless its long-term stability can be determined. Prospective use of **G** is potentially less sensitive to such variations when predicting short term selection responses. Our analysis provides a good illustration of the prospective versus retrospective usage of a G-matrix when considering the changes in **G**’s structure between **G**_control_ and **G**_tall_ and the respective predictions and inferences we can make from them.

## Conclusion

Our study showed that even highly plastic chemical traits such as floral scent, can be successfully included into predictive models of floral trait evolution. Even more so, we show that a complementary set of traits is important to consider, because pollinator selection acts on multiple traits, and genetic correlations link them in their evolutionary response. In the future, improved sampling and analysis techniques may allow the standard inclusion of a large set of traits and large sample sizes into evolutionary studies. Larger sample sizes may allow for more accurate predictions by incorporating the dynamics of G-matrix evolution over multiple generations. In addition, more assessments of selection on those traits in nature by specific groups of interacting organisms [21, 23, 24, 30, 61] may further improve our ability to predict evolutionary changes in the face of environmental change in natural habitats.

## Methods

### Plant species and focal traits

In our experiment, we used the lab-standard rapid cycling accession of *Brassica rapa* L. (syn. *B. campestris*: Brassicaceae) obtained from the Wisconsin Fast Plants™ Program (Carolina Biological Supply Company, Burlington, NC, USA). The rapid cycling accession was selected for short generation time, rapid seed maturation, absence of seed dormancy, small plant size and high female fertility [62]. *Brassica rapa* is generally recognized as a self-incompatible species with a generalized pollination system (e.g. bees, syrphid flies and butterflies as pollinators). However, the level of self-compatibility of this breed can evolve under selection [25]. The line used needs only ca. 35 to 40 days to complete a life cycle and maintains sufficient genetic variability for selection experiments [14, 15, 63, 64]. No specimen was deposited by us in a herbarium.

Our analysis includes a total of 16 traits with 12 floral volatile organic compounds (VOCs), and 4 morphological traits (plant height, petal width, petal length, and flower diameter). The measurement methods were described in detail in Zu & Schiestl [14]. Floral VOCs were collected from at least four freshly opened flowers per plant at a flow rate of 100 mL per min for 3 h. Floral VOC amounts were standardized in amounts per flower per liter sampled air, and ln(x + 1) transformed to approach normal distributions and z-scored (mean = 0, SD = 1) to normalize differences in scale between generations. Scent collection and analysis details can be found in Supporting information. The whole experiment was conducted at the Botanical Garden of the University of Zürich.

### Experiment I: artificial selection experiment

Details of the experimental procedure for artificial selection can be found in Zu & Schiestl [14]. To summarize, we sowed out 150 seeds to form the parental generation. Up and down directional artificial selection on plant height were imposed to produce a tall and a short line with the ten tallest and ten shortest plants, respectively. Additionally, ten randomly selected plants were chosen to form a control line. Selected plants were randomly hand pollinated within each line. Pollen donor, pollen receiver and their offspring were labeled for each fruit to generate a breeding pedigree. After fruit maturation, around 50 seeds from each of the three lines were sown out to form the next generation. The same procedures were carried out to obtain three generations of selection. Extra seeds were sowed out to ensure a minimum of 150 individual plants in each generation. In total, we analyzed 628 plants. The experiment was conducted in a phytotron with 24 h fluorescent light per day, 22 °C, 60% relative humidity, and regular watering twice a day (at 08:00 and 18:00).

### Experiment II: pollinator selection experiments

The procedures of experimental evolution experiment can be found in detail in Gervasi & Schiestl [25]. To summarize, we sowed out 300 seeds to generate 108 full sib families by manual cross pollination. These 108 full sib families were then equally divided into three replicates each containing 36 plants, for each of the three treatments (bumblebee, hoverfly, and hand pollination treatment). In each replicate, the 36 plants were placed in a 6*6 array with a distance of 20 cm from each other in a flight cage (2.5 m*1.8 m*1.2 m). In bumblebee and hoverfly treatments, five pollinators (either *Bombus terrestris* or *Episyrphus balteatus*) were introduced one at a time in the flight cage, with each allowed to freely visit maximal three different plants before being removed from the cage. A total number of 12–15 out of 36 plants per replicate received one or more pollinator visitation. The average (± s.d.) visitation (in visited plants) was 1.35 ± 0.63 for bumblebee-pollinated plants and 1.28 ± 0.53 for hoverfly-pollinated plants. In the control treatment 12 plants were randomly chosen and were manually pollinated among each other. Floral traits were measured prior to pollinators’ visits or hand pollination. The number of seeds were recorded after fruit maturation. Seeds from the pollinated plants were sown out proportionally (36/(replicate sum of seeds/individual seed set), values below 0.5 were rounded up to 1) to form again a total number of 36 plants for the next generation of each replicate. The same selection and sowing-out procedures were conducted for 9 generations, after which plants were sowed out again and randomly hand crossed between the replicates within each treatment to get rid of potential inbreeding depression. Fruits from random crosses were sown out to form the 11th generation and the measurements of floral traits in this generation were used as observed responses to selection.

### Estimation of the genetic variance-covariance matrices (G-matrix)

With known breeding pedigree and plant trait values for each individual in the control and treatment lines of the artificial selection experiment, we were able to estimate three genetic variance-covariance matrices: **G**_control_ in control, **G**_tall_ (or **G**_short_) in selection lines for increased (or decreased) plant height (see Table S3). The pedigree of the seeds sowed in the pollinator experiment was unknown. We thus used **G**_control_ from artificial selection experiment for evolutionary predictions in both experiments. More specifically, we estimated the G-matrix of the 16 traits by using a multivariate animal model in which the kinship (relatedness) matrix was obtained from the four-generation pedigree of the plants crossed within the experiments (sire = pollen donor, dam = pollen receiver), independently in the control, tall, and short experimental lines (Table S3). We fitted a linear mixed model using the Bayesian method implemented in the MCMCglmm R package [65] to estimate random effect variance components for additive genetic effects (V_A_) from which we estimated the G-matrix, and among-dam (V_D_) and among-sire (V_S_) components to remove potential maternal and paternal effects, respectively. We added generation as a block factor modeled as a fixed effect. This method was previously shown to have good applications with a few traits [50, 66].

In MCMCglmm, we used weakly informative inverse-Wishart prior with limit variance of one and covariance of zero and low degree of belief (0.002). Posterior distributions were robust to several different prior settings (e.g. V = diag(n)*0.1, V = diag(n)*10, n = number of traits). We used 1,200,000 iterations, with a burn-in of 200,000 and a thinning of 500 to ensure convergence and low autocorrelation among thinned samples (< 0.1). The thinning resulted in a posterior distribution with 2000 samples.

Finally, because the Bayesian approach does not allow us to directly test for the accuracy of our estimates of the G-matrices, we implemented a permutation test in which we randomly shuffled the dam and sire of each offspring within each generation and re-estimated the G-matrix for each of 500 replicates using the same MCMCglmm procedure as before. To evaluate the accuracy of the observed G-matrices (**G**_control_, **G**_tall_, and **G**_short_), we then compared them to their randomized estimates, element by element. For each element, we computed an empirical *P-*value as: *P = (N*_*random.estimates < observed.value*_*)/500*. If the observed value was smaller than the mean of the random estimates, then (1 – *P*) was used instead of *P*. The random estimates were obtained from the posterior mode of the 500 random estimates of each G-matrix. An element of **G** (a variance or covariance term within **G**) was considered significant if its *P*-value was < 0.05. If it was not the case, then the specific element estimation did not capture its biological meaning.

### Estimation of selection gradients

In the artificial selection experiment, we calculated the selection gradient on height (*β*_*h*_) by using

$$ {\beta}_h=S/{V}_{\mathrm{P}}, $$

where *V*_P_ is the phenotypic variation of height and *S* the selection differential calculated as the difference between the mean plant height of the selected plants and all measured plants in the same generation.

We calculated *β*_*h*_ in each generation and each selected line (Table S1) and used its sum over the three generations to predict the total evolutionary responses in each line.

In the pollinator selection experiments, we estimated the selection gradients following the partial correlation approach of Lande and Arnold [32]. To this end, we used a multi-linear regression model with relative seed set as dependent variable, replicate as factor and morphological and scent variables as covariates. Relative seed set was calculated as total number of seeds produced by a plant, divided by the mean number of seeds produced by all plants in the replicate. The regression coefficient estimates (selection gradients) were obtained from the multi-linear regression. The selection gradients (**β**) were calculated separately per treatment (bumblebee and hoverfly), for all the measured generations and replicates combined (for details, see [25]). The non-significant selection gradients were still used as the best approximate estimations of selection.

### Calculation of predicted and observed evolutionary changes

To estimate the predicted responses to selection, we used the multivariate breeder’s equation [31], △**z** = **Gβ** (see Introduction). We used **G** from the control group in the artificial selection experiment (**G**_control_, Table S3, S4) for predictions as the best estimation of genetic architecture of the original population. We used the 2000 posterior samples of the G-matrix to generate a distribution of predicted trait changes from which we could evaluate the accuracy of our evolutionary prediction using its 95% highest posterior density (HPD) interval.

To calculate the observed trait changes, we calculated the observed phenotypic changes between the last and the first generation (△z_obs. = *X*_Fn_ - *X*_P_, where n is 3 in artificial selection experiment, and 11 in pollinator selection experiment) for each line or each treatment, and P stands for ‘parental’ (generation 1 in Control condition in the pollinator selection experiment). We present the observed and predicted changes scaled by the phenotypic standard deviation of each trait in the parental generation.

### Direct and indirect selection responses

To examine the importance of trait covariance in affecting evolutionary trajectories, we separated the total selection response △**z** of each trait into its direct and indirect components. The direct component of the predicted selection response of trait *i* is the product G_ii_*β_*i*_, with G_*ii*_ the additive genetic variance of the trait (diagonal element of **G**_control_). The indirect component is the product of the off-diagonal elements of **G** (genetic covariance: *G*_*ij*_) with **β**, summed over all traits *j* ≠ *i*: △*z*_*i*_^indirect^ = ∑*G*_ij_*β_*j*_. The total response is the sum of these two components. The three predictions, indirect, direct, and total response were compared to the observed change of each trait to evaluate when the direct response is constrained (direct and indirect components of opposite sign) or enhanced (direct and indirect components of same sign) by genetic covariance.

Finally, we measured the constraining effect of genetic co-variation on the response to selection by comparing the angle **θ** between the selection response vector (△**z**) and the first PC of **G** (PC1, or ***g***_max_) with the angle **γ** between △**z** and the selection gradient (**β**). We generated the posterior distribution of **θ** from the posterior distribution of **G**_control_, which allowed us to test whether **γ** is larger (smaller) than **θ**, which tests if △**z** is biased (unbiased) in the direction of ***g***_max_ by genetic correlations.

### G-matrices similarity among artificial selection lines

We compared the **G**-matrices from control, tall and short selection lines to assess the stability of **G** between treatments and control. We used the random skewers (RS) method in one comparison test because it examines the similarity between two **G**-matrices of their expected evolutionary response to a random set of selection vectors (skewers), which fits our purpose of evaluating the stability of such predictions using different estimates of the **G**-matrix. We used Roff et al.’s (2012) implementation of the RS method, and report the mean over 10,000 random selection skewers of the correlation between the selection response vectors of the two **G**-matrices compared. Significance was obtained from the distribution of the test statistics obtained from the 500 random estimates of each **G**-matrix. We performed a further test of shape similarity between the **G**-matrices using the hierarchical approach of Phillips and Arnold [37], also known as the Flury hierarchy, implemented in Roff et al.’s R script collection [67]. This method tests the degree of shape similarity sequentially by comparing the size and orientation of the eigenvectors (principal components, PCs) of the **G**-matrices. Two **G**-matrices can have common principal components (CPC) if their PCs have the same orientation but not the same size (i.e., have different eigenvalues), be proportional if their PCs only differ proportionally, or be equal. The three levels of similarity are tested relative to the hypothesis of unrelated matrices. The test statistics are provided in Roff et al. [67]. We determined the significance of the RS and Flury tests using the previous 500 randomized estimates of **G**_control_, **G**_tall_, and **G**_short_.

All statistics were conducted with R version 3.3.3 [68]..

## Supplementary information

**Additional file 1. **Additional methods: ***Scent collection and analysis.*** Additional methods: ***Comparing shape, size and orientation of G-matrices.*** **Table S1.** Selection gradients on plant height. **Table S2.** Linear and quadratic selection gradients in bumblebee and hoverfly experiments, and control lines. **Table S3.** Model setups for MCMCglmm analyses of G-matrices estimation. **Table S4.** G-matrix estimates in Control, Tall, and Short lines. **Table S5.** Eigen-structure (eigenvalues and PC1) of posterior G-matrices. **Table S6.** Observed and predicted trait changes in the bumblebee and hoverfly experiments.

## Data Availability

The phenotypic data and R scripts are available at 10.5281/zenodo.4022510.

## Abbreviations

VOC: Volatile organic compounds
HPD: High probability density
PW: Petal width
PL: Petal length
FD: Flower diameter
Ben: Benzaldehyde
PAA: Phenylacetaldehyde
FAR: α-Farnesene
BenN: Benzyl nitrile
Aben: 2-Amino benzaldehyde
Ind: Indole
MA: Methyl anthranilate
PA: Phenylethyl alcohol
MS: Methyl salicylate
MB: Methyl benzoate
ZHA: Z-(3)-Hexenyl acetate
ITC: 1-Butene-4-isothiocyanate
