# Supplementary material for: Floral signals evolve in a predictable way under artificial and pollinator selection in Brassica rapa
Source: BMC Evol Biol. 2020 Sep 24;20:127. doi: 10.1186/s12862-020-01692-7 (PMC7517814; doi:10.1186/s12862-020-01692-7)
Supplement: Supplementary file 1 — Additional file 1. Additional methods: Scent collection and analysis. Additional methods: Comparing shape, size and orientation of G-matrices. Table S1. Selection gradients on plant height. Table S2. Linear and quadratic selection gradients in bumblebee and hoverfly experiments, and control lines. Table S3. Model setups for MCMCglmm analyses of G-matrices estimation. Table S4. G-matrix estimates in Control, Tall, and Short lines. Table S5. Eigen-structure (eigenvalues and PC1) of posterior G-matrices. Table S6. Observed and predicted trait changes in the bumblebee and hoverfly experiments. [file 12862_2020_1692_MOESM1_ESM.pdf]

Floral signals evolve in a predictable way under artificial and pollinator selection in *Brassica rapa*

Pengjuan Zu, Florian P. Schiestl, Daniel Gervasi, Xin Li, Daniel Runcie, Frédéric Guillaume

Supporting Information

Methods

Scent collection and analysis

The measurement methods were described in detail in Zu & Schiestl (2017). We collected floral volatiles of the entire inflorescence, when at least four freshly opened flowers were present, by using a push-pull system, and analyzed floral VOCs by using a gas chromatograph with a mass-selective detector (GC-MSD) fitted with a thermal desorption system (Gerstel TDS/TDU, Mülheim an der Ruhr, Germany). With the push-pull scent collection system, the whole inflorescence was fixed with two Teflon plates (with an opening in the middle for the stem) and was covered in a glass cylinder with silanized (deactivated) surfaces (Sigmacote, Sigma, MO, USA). Two glass ports were used to push-in clean air and pull-out air with volatiles for sampling. Through the push port we introduced a filter with activated charcoal (SKC Eighty Four, PA, USA) which was connected to an air pump to push purified air at 100 mL per min into the glass cylinder. Through the pull port we introduced a glass tube filled with adsorbent (35 mg Tenax TA 60/80, Supelco, Bellefonte, PA, USA) which was connected to a vacuum pump for pulling out air containing inflorescence VOCs at a flow rate of 100 mL per min, for 3 hours for each plant. Wisconsin's rapid cycling *B. rapa* has been bred under 24 hours light a day. No significant daily dynamics of scent emission were found in a pilot experiment (scent collection for 3 plants during three different time periods of a day, data not shown). Thus, we collected scent during the following time periods: 07:00 – 10:00, 12:00 – 15:00, and 17:00 – 20:00 hours, depending on the number of available plants. At least one air control from an empty glass cylinder was sampled in each collection period.

To analyze floral VOCs, each sample was injected into a gas chromatograph (GC, Agilent 6890N, Agilent Technologies, Palo Alto, CA, USA) using a Gerstel Multipurpose Sampler. For thermal desorption, a temperature program was run, starting at 30°C (1 minute

hold) increasing to 240°C (1 minute hold) at 60°C min^{-1}. A cool injection system (CIS 4, Gerstel Germany) was used for cryotrap enrichment of eluting volatiles from the TDS. The CIS started with a temperature of -150°C (0.05 minute hold) and increased to 150°C at 16°C s^{-1}, then increased further from 150°C to 250°C at 12°C s^{-1}. The GC was equipped with an HP-5 capillary column (Agilent, 15m length, 0.25mm diameter, 0.25µm film thickness). The temperature of the GC oven was set to 50°C (1 minute hold) and increased to 250°C at 10°C min^{-1}, and helium was used as the carrier gas, with a constant flow of 2 ml min^{-1}. An Agilent 5975 Series MSD mass spectrometer was used for compound identification and quantification.

We used an Agilent 5975 Series MSD mass spectrometer for compound identification and quantification. To identify volatile compounds, mass spectra obtained from the samples were compared with those of a reference collection (NIST, the National Institute of Standards and Technology, mass spectral library). Subsequently, retention times and mass spectra of all compounds included in the quantitative analyses were compared with those of synthetic reference standards. Compound quantification was carried out with the ChemStation Enhanced Data Analysis program (Version E.01.00). For all compounds included in the study, synthetic standards were analyzed in three different amounts using the GC-MSD (1, 10 and 100 ng). By using target ions for each compound, calibration curves were established using the ChemStation program. Peak areas of target ions were subsequently used for quantifying the amounts of volatiles in the samples.

Comparing shape, size and orientation of G-matrices

Besides the random skewer method to compare the similarity of G-matrices, we further evaluated the size, the shape, and the orientation of the G-matrices (Puentes *et al.*, 2016) in the control-, tall-, and short-lines by examining their posterior G-matrices obtained from *MCMCglmm* models.

First, the shape of the G-matrices were compared by using a principal component (PC) analysis to characterize the main axes and the loadings of the traits on the PCs. We calculated the eigenvectors and eigenvalues (λ_i, i=1, 2, 3,…, p) by performing singular value decomposition (SVD) of G-matrix(Golub & van Loan, 1983) using the *eigen* function in R v3.3.3.

Second, we used the orientation of the first important PC (PC1) as representative for the major orientation of a G-matrix ($\boldsymbol{g}_{max}$). We examined the angles of PC1s within and between

posterior distributions of the four **G**s by using the equation of angles between two non-zero vector *u* and *v*: $cos\theta = \frac{u \cdot v}{\|u\| \|v\|}$.

References

Golub G, van Loan C. 1983. Measuring Vectors, Matrices, Subspaces, and Linear System Sensitivty. *Chapter* **2**: 10-30.

Puentes A, Granath G, Ågren J. 2016. Similarity in G matrix structure among natural populations of Arabidopsis lyrata. *Evolution* **70**(10): 2370-2386.

Table S1. Selection gradients on plant height in *Brassica rapa* in the tall and short artificial selection experiment for three generations.

	**Tall**	**Short**
**β1**	0.3543	-0.2828
**β2**	0.3313	-0.2153
**β3**	0.2914	-0.3872
**Total**	0.9770	-0.8853

Table S2. Linear selection gradients and standard error on morphological and scent traits in *Brassica rapa* in bumblebee, hoverfly selection and control groups. We used pooled data from all the generations and all replicates in each experiment, with replicate as random factor and a Poisson error distribution.

	Bumblebee		Hoverfly	
	Linear selection		Linear selection	
**Traits**	β	SE	β	SE
**Plant height**	0.587	0.1155***	0.303	0.1246*
**Petal width**	0.064	0.1673	0.159	0.1678
**Petal length**	0.052	0.1291	0.060	0.1346
**Flower diameter**	0.220	0.1676	0.123	0.1642
**Benzaldehyde**	-0.005	0.1165	-0.105	0.1343
**Phenylacetaldehyde**	0.188	0.1806	-0.258	0.1710
**(E)-α-Farnesene**	-0.031	0.1184	0.191	0.1207
**Benzyl nitrile**	-0.237	0.1982	0.118	0.1698
**2-Amino benzaldehyde**	0.013	0.1628	-0.039	0.1459
**Indole**	0.097	0.1817	0.236	0.1726
**Methyl anthranilate**	-0.059	0.1838	-0.329	0.1649*
**Phenylethyl alcohol**	-0.207	0.1571	-0.114	0.1482
**Methyl salicylate**	0.025	0.1359	0.070	0.1325
**Methyl benzoate**	0.215	0.1594	-0.029	0.1542
**Z-(3)-Hexenyl acetate**	-0.101	0.1110	-0.044	0.1327
**1-Butene-4-isothyocy.**	-0.051	0.1076	0.051	0.1245

Table S3. Different model setups for MCMCglmm analyses of G-matrices estimation. Models include the three models from different lines throughout the artificial selection experiment in *Brassica rapa*. Generation 0, parental generation; 1, 2, 3, offspring generations. N, plant sample size for each model.

G names	Line	Generation	Fixed factor	Random factor	N
$G_{control}$	Control	0,1,2,3	Generation	Dam, sire	298
G_{tall}	Tall	0,1,2,3	Generation	Dam, sire	298
G_{short}	Short	0,1,2,3	Generation	Dam, sire	290

Table S4. G-matrix estimates in Control, Tall, and Short lines. Posterior mode of genetic variance (diagonal), covariance (lower triangular) and genetic correlations (upper triangular) estimated from control, tall, and short lines in the artificial selection experiment. Significant values are bolded (uncorrected values).

	**Height**	**PW**	**PL**	**FD**	**Ben**	**PAA**	**FAR**	**BenN**	**Aben**	**Ind**	**MA**	**PA**	**MS**	**MB**	**ZHA**	**ITC**
Control																
Height	**23.192**	**0.142**	**0.272**	**0.437**	**0.165**	**0.275**	**0.282**	**0.113**	**0.285**	**0.322**	**0.311**	**0.296**	**0.054**	**0.184**	**0.190**	**0.139**
PW	**0.369**	**0.292**	**0.292**	**0.380**	**0.051**	**0.155**	0.082	**0.116**	**0.141**	**0.122**	**0.154**	**0.173**	0.076	**0.088**	**0.020**	-0.017
PL	**0.704**	**0.085**	**0.288**	**0.521**	**0.030**	**0.171**	**0.163**	**0.131**	**0.166**	**0.190**	**0.230**	**0.142**	0.034	**0.139**	**0.045**	0.031
FD	**1.978**	**0.193**	**0.263**	**0.881**	**0.033**	**0.245**	**0.165**	**0.156**	**0.245**	**0.254**	**0.294**	**0.286**	**0.088**	**0.167**	**0.080**	**0.117**
Ben	**0.493**	**0.017**	**0.010**	**0.019**	0.384	**0.163**	**0.152**	**0.141**	**0.228**	**0.176**	**0.103**	**0.069**	0.057	**0.204**	0.117	**0.053**
PAA	**1.297**	**0.082**	**0.090**	**0.226**	**0.099**	**0.963**	**0.244**	**0.360**	**0.412**	**0.324**	**0.329**	**0.664**	**0.243**	**0.202**	**0.132**	**0.190**
FAR	**0.744**	0.024	**0.048**	**0.085**	**0.052**	**0.131**	**0.300**	**0.196**	**0.295**	**0.246**	**0.262**	**0.273**	0.197	**0.149**	0.141	**0.192**
BenN	**0.328**	**0.038**	**0.042**	**0.088**	**0.053**	**0.213**	**0.065**	**0.364**	**0.325**	**0.368**	**0.269**	**0.428**	0.100	**0.200**	0.047	**0.115**
Aben	**1.264**	**0.070**	**0.082**	**0.212**	**0.130**	**0.372**	**0.149**	**0.181**	**0.848**	**0.631**	**0.481**	**0.324**	**0.152**	**0.156**	**0.151**	**0.160**
Ind	**1.100**	**0.047**	**0.072**	**0.169**	**0.077**	**0.225**	**0.096**	**0.157**	**0.412**	**0.502**	**0.399**	**0.363**	**0.234**	**0.139**	**0.181**	**0.205**
MA	**1.215**	**0.067**	**0.100**	**0.224**	**0.052**	**0.262**	**0.116**	**0.132**	**0.360**	**0.229**	**0.658**	**0.348**	**0.300**	**0.429**	0.117	0.096
PA	**1.077**	**0.070**	**0.057**	**0.202**	**0.032**	**0.492**	**0.113**	**0.195**	**0.225**	**0.194**	**0.213**	**0.569**	**0.253**	**0.266**	**0.190**	**0.289**
MS	**0.197**	0.032	0.014	**0.063**	0.027	**0.182**	0.082	0.046	**0.106**	**0.126**	**0.185**	**0.145**	**0.581**	**0.314**	0.124	0.035
MB	**0.624**	**0.034**	**0.052**	**0.111**	**0.089**	**0.140**	**0.058**	**0.085**	**0.101**	**0.069**	**0.246**	**0.142**	**0.169**	**0.498**	**0.181**	**0.199**
ZHA	**0.586**	**0.007**	**0.015**	**0.048**	0.047	**0.083**	0.049	0.018	**0.089**	**0.082**	0.061	**0.092**	0.061	**0.082**	**0.412**	**0.169**
ITC	**0.579**	-0.008	0.014	**0.095**	**0.028**	**0.162**	**0.091**	**0.060**	**0.128**	**0.126**	0.067	**0.190**	0.023	**0.122**	**0.094**	0.753
Tall																
Height	**2.075**	0.021	**0.171**	**0.272**	0.086	0.027	0.039	**-0.106**	0.018	0.068	0.130	**0.119**	-0.058	**0.121**	0.069	-0.040
PW	0.016	0.293	**0.291**	0.334	**0.111**	0.073	0.071	**0.139**	0.083	0.021	**0.125**	**0.101**	0.075	**0.092**	**0.030**	-0.103
PL	**0.132**	**0.085**	**0.287**	**0.546**	0.053	0.091	**0.056**	**0.143**	**0.120**	0.091	0.110	0.079	**0.071**	**0.109**	-0.031	-0.006
FD	**0.360**	0.166	**0.269**	**0.846**	**0.057**	**0.221**	**0.106**	**0.133**	0.070	0.078	0.101	**0.138**	**0.101**	**0.091**	**0.038**	-0.090
Ben	0.076	**0.037**	0.017	**0.032**	**0.378**	0.054	0.150	0.112	**0.227**	**0.150**	**0.103**	**0.129**	**0.117**	**0.296**	**0.222**	0.026
PAA	0.033	0.034	0.042	**0.173**	0.028	**0.726**	**0.190**	**0.341**	**0.312**	**0.238**	**0.268**	**0.574**	**0.117**	0.051	0.010	0.079
FAR	0.030	0.021	**0.016**	**0.053**	0.050	**0.088**	**0.295**	0.149	**0.235**	**0.244**	0.159	**0.202**	**0.220**	**0.138**	**0.144**	0.136
BenN	**-0.089**	**0.044**	**0.044**	**0.071**	0.040	**0.169**	0.047	**0.337**	**0.338**	**0.337**	**0.206**	**0.289**	**0.188**	**0.115**	**0.129**	0.009
Aben	0.020	0.035	**0.050**	0.051	**0.110**	**0.208**	**0.100**	**0.154**	0.615	**0.523**	0.439	**0.263**	**0.182**	**0.100**	0.103	0.103
Ind	0.066	0.008	0.033	0.048	**0.062**	**0.137**	**0.089**	**0.132**	**0.276**	**0.453**	**0.370**	**0.170**	**0.152**	**0.049**	0.093	0.109

MA	0.139	**0.050**	0.044	0.069	**0.047**	**0.170**	0.064	**0.089**	0.256	**0.185**	**0.553**	**0.211**	**0.274**	**0.336**	**0.161**	**0.162**
PA	**0.115**	**0.037**	0.029	**0.086**	**0.053**	**0.329**	**0.074**	**0.113**	**0.139**	**0.077**	**0.106**	**0.452**	**0.125**	**0.200**	0.128	**0.205**
MS	-0.063	0.030	**0.028**	**0.069**	**0.053**	**0.074**	**0.089**	**0.081**	**0.106**	**0.076**	**0.152**	**0.063**	**0.553**	**0.358**	0.063	**0.050**
MB	**0.120**	**0.034**	**0.040**	**0.058**	**0.125**	0.030	**0.052**	**0.046**	**0.054**	**0.023**	**0.172**	**0.093**	**0.183**	**0.474**	0.200	0.074
ZHA	0.058	**0.009**	-0.010	**0.020**	**0.079**	0.005	**0.045**	**0.043**	0.047	0.037	**0.069**	0.050	0.027	0.080	0.337	**0.133**
ITC	-0.047	-0.045	-0.003	-0.068	0.013	0.055	0.061	0.004	0.066	0.060	**0.099**	**0.113**	**0.030**	0.042	**0.063**	0.669
Short																
Height	23.163	0.119	0.247	0.254	**0.370**	**0.235**	0.222	**0.216**	**0.437**	**0.448**	**0.387**	**0.194**	0.011	**0.306**	0.274	**0.118**
PW	0.326	**0.323**	**0.282**	**0.335**	**0.092**	**0.140**	0.034	**0.136**	**0.097**	**0.114**	**0.168**	**0.091**	0.056	**0.125**	0.031	**-0.008**
PL	0.649	**0.087**	**0.298**	**0.560**	**0.082**	**0.196**	**0.078**	0.094	**0.158**	**0.166**	**0.166**	**0.068**	0.019	**0.085**	**0.064**	-0.029
FD	1.098	**0.171**	**0.275**	**0.809**	0.045	**0.214**	**0.087**	**0.120**	**0.258**	**0.189**	**0.241**	**0.221**	-0.014	**0.129**	**0.102**	**0.018**
Ben	**1.149**	**0.034**	**0.029**	0.026	**0.417**	**0.155**	**0.210**	0.076	**0.289**	0.193	**0.188**	0.096	**0.176**	**0.360**	**0.245**	0.070
PAA	**1.048**	**0.074**	**0.099**	**0.178**	**0.093**	**0.861**	**0.227**	0.311	**0.276**	**0.308**	**0.302**	**0.609**	0.123	**0.231**	**0.148**	**0.145**
FAR	0.822	0.015	**0.033**	**0.060**	**0.104**	**0.162**	**0.592**	0.102	**0.210**	**0.240**	**0.176**	0.152	**0.154**	0.067	**0.145**	0.119
BenN	**0.603**	**0.045**	0.030	**0.063**	0.028	0.167	0.046	**0.335**	**0.330**	**0.303**	**0.268**	0.248	0.149	**0.169**	**0.117**	0.060
Aben	**1.783**	**0.047**	**0.073**	**0.196**	**0.158**	**0.217**	**0.137**	**0.162**	**0.718**	**0.539**	**0.565**	**0.246**	0.130	**0.194**	**0.171**	**0.140**
Ind	**1.468**	**0.044**	**0.062**	**0.116**	0.085	**0.195**	**0.126**	**0.120**	**0.311**	**0.464**	**0.428**	**0.255**	0.162	**0.191**	**0.223**	**0.138**
MA	**1.509**	**0.077**	**0.073**	**0.176**	**0.099**	**0.227**	**0.110**	**0.126**	**0.388**	**0.237**	**0.658**	**0.238**	**0.201**	**0.430**	**0.332**	**0.129**
PA	**0.666**	**0.037**	**0.026**	**0.142**	0.044	**0.404**	0.083	0.103	**0.149**	**0.124**	**0.138**	**0.511**	0.108	**0.255**	**0.170**	0.088
MS	0.039	0.023	0.008	-0.009	**0.085**	0.085	**0.088**	0.064	0.082	**0.082**	**0.121**	0.058	**0.553**	0.259	0.120	**0.090**
MB	**0.992**	**0.048**	**0.031**	**0.078**	**0.156**	**0.144**	0.035	**0.066**	**0.111**	**0.088**	**0.235**	**0.123**	0.130	**0.452**	**0.302**	0.131
ZHA	0.933	0.012	**0.025**	**0.065**	**0.112**	**0.097**	**0.079**	**0.048**	**0.102**	**0.108**	**0.191**	**0.086**	0.063	**0.144**	**0.501**	**0.221**
ITC	**0.528**	**-0.004**	-0.015	**0.015**	0.042	**0.125**	0.085	0.032	**0.110**	**0.088**	**0.097**	0.058	**0.062**	0.082	**0.145**	**0.865**

Table S5. Eigen-structure (eigenvalues and PC1) of posterior G-matrices.

	Eigenvalues			PC1		
	$\mathbf{G}_{control}$	$\mathbf{G}_{tall}$	$\mathbf{G}_{short}$	$\mathbf{G}_{control}$	$\mathbf{G}_{tall}$	$\mathbf{G}_{short}$
Height	23.7743	2.7994	22.3011	0.9863	0.9687	0.9835
PW	2.1650	1.4876	1.7313	0.0235	0.0447	0.0205
PL	1.0032	0.9289	1.0776	0.0300	0.0581	0.0290
FD	0.7969	0.7957	0.7964	0.0881	0.1254	0.0616
Ben	0.6700	0.6059	0.6578	0.0250	0.0400	0.0439
PAA	0.5268	0.5207	0.5439	0.0640	0.0612	0.0614
FAR	0.4185	0.4199	0.4438	0.0381	0.0306	0.0442
BenN	0.3622	0.3452	0.3798	0.0154	0.0005	0.0217
Aben	0.3142	0.3101	0.3415	0.0705	0.1147	0.0888
Ind	0.2613	0.2570	0.2910	0.0464	0.0388	0.0659
MA	0.2444	0.2326	0.2548	0.0539	0.1161	0.0776
PA	0.2133	0.2041	0.2260	0.0493	0.0749	0.0291
MS	0.1889	0.1793	0.2048	0.0162	0.0384	0.0235
MB	0.1698	0.1663	0.1783	0.0355	0.0708	0.0512
ZHA	0.1518	0.1480	0.1559	0.0279	0.0112	0.0476
ITC	0.1279	0.1263	0.1319	0.0317	-0.0478	0.0427

Table S6. Observed and predicted trait changes in the (A) Bumblebee and (B) Hoverfly experiments. The predicted changes are obtained from the multivariate breeder's equation and are decomposed into the direct and indirect components of the total response to selection (see main text). Figures in bold are predicted responses who's 95% HPD interval does not overlap with zero or observed changes who's value falls within the 95% HPD interval of its total predicted value. Values in italic are traits whose observed change and direct response are in the same direction.

	**Observed changes**	**Predicted changes**								
		**Total**	95% HPD		**Direct**	95% HPD		**Indirect**	95% HPD	
			lower	upper		lower	upper		lower	upper
(A) Bumblebee										
Plant height (Height)	***2.082***	**3.004**	2.032	3.790	**2.877**	1.928	3.645	**0.103**	0.036	0.201
Petal width (PW)	-0.064	**0.706**	0.089	1.096	**0.029**	0.022	0.039	**0.675**	0.064	1.063
Petal length (PL)	-0.897	**0.889**	0.245	1.411	**0.027**	0.020	0.035	**0.861**	0.225	1.386
Flower diameter (FD)	*0.039*	**0.914**	0.510	1.415	**0.130**	0.094	0.190	**0.763**	0.389	1.247
Benzaldehyde (Ben)	*-0.884*	0.511	-0.386	1.407	**-0.004**	-0.006	-0.003	0.516	-0.381	1.412
Phenylacetaldehyde (PAA)	***1.046***	**0.722**	0.059	1.328	**0.152**	0.100	0.235	0.604	-0.074	1.158
α-Farnesene (FAR)	**0.292**	**0.613**	0.237	1.226	**-0.013**	-0.018	-0.010	**0.713**	0.252	1.246
Benzyl nitrile (BenN)	1.049	0.203	-0.322	0.680	**-0.098**	-0.130	-0.070	0.245	-0.223	0.787
2-Amino benzaldehyde (Aben)	***0.920***	**0.568**	0.068	1.032	**0.007**	0.004	0.010	**0.562**	0.058	1.021
Indole (Ind)	***1.179***	**0.751**	0.187	1.242	**0.046**	0.032	0.065	**0.706**	0.154	1.193
Methyl anthranilate (MA)	**0.961**	**0.554**	0.033	1.327	**-0.039**	-0.055	-0.025	**0.586**	0.054	1.356
Phenylethyl alcohol (PA)	**0.254**	**1.158**	0.014	2.498	**-0.247**	-0.398	-0.188	**1.467**	0.232	2.803
Methyl salicylate (MS)	-0.207	0.174	-0.432	0.903	**0.018**	0.012	0.024	0.149	-0.450	0.881
Methyl benzoate (MB)	***0.701***	**0.753**	0.058	1.359	**0.140**	0.100	0.189	0.595	-0.101	1.174
Z-(3)-Hexenyl acetate (ZHA)	0.204	0.452	-0.189	0.987	**-0.055**	-0.073	-0.040	0.324	-0.128	1.049
1-Butene-4-isothiocyanate (ITC)	0.491	0.510	-0.628	1.605	**-0.054**	-0.087	-0.036	0.516	-0.635	1.623

	**Observed changes**	**Predicted changes**								
		**Total**	95% HPD		**Direct**	95% HPD		**Indirect**	95% HPD	
			lower	upper		lower	upper		lower	upper
(B) Hoverfly										
Plant height (Height)	*0.381*	**1.486**	1.008	1.921	**1.485**	0.995	1.881	-0.018	-0.108	0.099
Petal width (PW)	-0.852	**0.295**	0.046	0.572	**0.072**	0.055	0.096	0.277	-0.013	0.500
Petal length (PL)	-1.225	**0.424**	0.139	0.749	**0.031**	0.023	0.040	**0.434**	0.108	0.715
Flower diameter (FD)	-0.339	**0.465**	0.215	0.689	**0.073**	0.053	0.106	**0.387**	0.146	0.595
Benzaldehyde (Ben)	*-0.548*	0.181	-0.320	0.595	**-0.087**	-0.117	-0.065	0.266	-0.242	0.677
Phenylacetaldehyde (PAA)	*-0.326*	0.087	-0.246	0.404	**-0.208**	-0.322	-0.138	0.258	-0.038	0.631
α-Farnesene (FAR)	***0.178***	**0.364**	0.107	0.616	**0.078**	0.062	0.109	**0.235**	0.046	0.538
Benzyl nitrile (BenN)	-0.059	0.049	-0.171	0.345	**0.049**	0.035	0.065	0.055	-0.215	0.306
2-Amino benzaldehyde (Aben)	*-0.309*	0.193	-0.049	0.441	**-0.022**	-0.029	-0.012	0.216	-0.025	0.467
Indole (Ind)	*0.021*	**0.308**	0.091	0.631	**0.113**	0.078	0.158	0.189	-0.034	0.485
Methyl anthranilate (MA)	0.059	0.179	-0.196	0.456	**-0.216**	-0.307	-0.137	0.346	-0.002	0.686
Phenylethyl alcohol (PA)	*-2.162*	0.362	-0.330	0.935	**-0.136**	-0.219	-0.103	0.449	-0.162	1.111
Methyl salicylate (MS)	-0.847	0.089	-0.271	0.408	**0.049**	0.034	0.068	-0.043	-0.336	0.343
Methyl benzoate (MB)	0.435	0.158	-0.141	0.526	**-0.019**	-0.025	-0.014	0.149	-0.118	0.553
Z-(3)-Hexenyl acetate (ZHA)	0.014	0.244	-0.145	0.450	**-0.024**	-0.032	-0.017	0.168	-0.121	0.476
1-Butene-4-isothiocyanate (ITC)	*0.694*	0.350	-0.311	0.849	**0.054**	0.036	0.087	0.206	-0.360	0.787
